# A deep ensemble learning method for single finger-vein identification

**DOI:** 10.3389/fnbot.2022.1065099

**Published:** 2023-01-11

**Authors:** Chongwen Liu, Huafeng Qin, Qun Song, Huyong Yan, Fen Luo

**Affiliations:** ^1^College of Artificial Intelligence, Chongqing Technology and Business University, Chongqing, China; ^2^Chongqing Key Laboratory of Intelligent Perception and BlockChain Technology, Chongqing Technology and Business University, Chongqing, China

**Keywords:** finger-vein recognition, single sample per person, deep learning, ensemble learning, pattern recognition

## Abstract

Finger-vein biometrics has been extensively investigated for personal verification. Single sample per person (SSPP) finger-vein recognition is one of the open issues in finger-vein recognition. Despite recent advances in deep neural networks for finger-vein recognition, current approaches depend on a large number of training data. However, they lack the robustness of extracting robust and discriminative finger-vein features from a single training image sample. A deep ensemble learning method is proposed to solve the SSPP finger-vein recognition in this article. In the proposed method, multiple feature maps were generated from an input finger-vein image, based on various independent deep learning-based classifiers. A shared learning scheme is investigated among classifiers to improve their feature representation captivity. The learning speed of weak classifiers is also adjusted to achieve the simultaneously best performance. A deep learning model is proposed by an ensemble of all these adjusted classifiers. The proposed method is tested with two public finger vein databases. The result shows that the proposed approach has a distinct advantage over all the other tested popular solutions for the SSPP problem.

## 1. Introduction

With the wide application of the internet, information security has become increasingly critical. Traditional personal identification technique, such as key and password, is difficult to meet people's needs. For example, the key is easily copied and missed, and the password is usually forgotten, especially for older people. Biometric technique as a solution has been widely investigated in recent years. Compared to the traditional identification approach, physiological characteristics to identify or verify a person has the following advantages (Albrecht et al., 2009): (1) Difficult to be missed; (2) Difficult to be forged; (3) Easy to use; (4) Easy to carry. Various traits, such as the face iris, fingerprint, and vein, have been employed for the recognition of a person and are broadly split into two categories (Vodinh, 2012; Kuzu et al., 2020): (1) Extrinsic characteristics, e.g., face, iris, and fingerprint. (2) Intrinsic characteristics, e.g., finger-vein, hand-vein, and palm-vein. Extrinsic characteristics are suspected to be copied and forged, and their fake version has been proven to be successfully employed to attack the recognition system (Khan et al., 2015), and improve the quality of service (Wu et al., 2022). On the contrary, the intrinsic characteristics are concealed in our bodies and are very difficult to be copied without the user's willingness. In addition, only the vein in a living body can be captured effectively and further used for identification. Thus, intrinsic traits provide high privacy and security in practical applications.

### 1.1. Related work

Despite the recent advances in finger-vein biometric recognition, it is still a challenging task in practical application since vein-capturing results are easily affected by many factors, such as illumination, environment temperature, and the behavior of the user. These factors cannot avoid during the capturing process, in this case, the training dataset may contain a large number of low-quality finger-vein images, which may decrease the recognition accuracy. To achieve robust recognition, various approaches are proposed for vein recognition in recent years and are broadly categorized into the following categories (Hou et al., 2022; Shaheed et al., 2022b).

(1) Local descriptor-based approaches: Local descriptor-based approaches mainly consist of local statistical information-based methods and local invariant-based methods. Local statistical information-based methods include local binary pattern (LBP) (Lee et al., 2010, 2011; Yang et al., 2014; Kang et al., 2015), local line binary pattern (LLBP) (Rosdi et al., 2011; Yang et al., 2013), efficient local binary pattern (ELBP) (Liu and Kim, 2016), discriminative binary codes (DBC) (Xi et al., 2016), and fuzzy images (Qin et al., 2022). A typical representation of local invariant-based methods is the scale-invariant feature transform (SIFT) (Qin et al., 2013; Wang et al., 2015).

(2) Superpixel-based feature extraction approach: The representative approaches in this category are the superpixel-based feature (Liu et al., 2014), vein textons map (Dong et al., 2014), hyper information feature (Xi et al., 2014), and personalized feature (Xi et al., 2013). Superpixel-based feature extraction methods have achieved a high recognition rate in some public databases (Kirchgasser et al., 2020).

(3) Subspace-learning-based approaches: Subspace learning as a powerful technique have been widely used in pattern recognition task such as vein identification. A projection matrix computed from training data is employed to map the finger-vein images into subspace, and the resulting features are further used for recognition. The typical methods include principal component analysis (PCA) (Wu and Liu, 2011a), two dimensional principal component analysis (2DPCA) (Qiu et al., 2016), two-directional and two-dimensional principal component analysis ((2D)2PCA) (Yang et al., 2012; Li et al., 2017; Zhang et al., 2021; Ban et al., 2022; She et al., 2022), linear discriminant analysis (LDA) (Wu and Liu, 2011b), high-dimensional state space (Zhang et al., 2022), self-feature-based method (Xie et al., 2022), and latent factor model (Wu et al., 2022).

(4) Deep learning-based approaches: The deep learning-based approaches have been directly used to learn robust features from original images and successfully applied for computation vision tasks. Some researchers brought them into finger-vein recognition. For example, deep learning approaches are employed for vein image segmentation (Liskowski and Krawiec, 2016; Qin et al., 2019; Yang et al., 2019; Shaheed et al., 2022a), quality assessment of vein image (Qin and Yacoubi, 2015; Qin and El-Yacoubi, 2018), fuzzy networks (Liu H. et al., 2022; Lu et al., 2022; Muthusamy and Rakkimuthu, 2022), and finger-vein recognition (Wang et al., 2017; Avci et al., 2019; Gumusbas et al., 2019; Zhang J. et al., 2019).

### 1.2. Motivation

As discussed in the related works, the handcrafted-based approaches are proposed based on prior human knowledge, some vein features related to recognition may be missed during the feature extraction process. On the contrary, the deep learning-based extraction approaches without any prior assumption can automatically extract high-level features by representation learning that are objectively related to vein recognition. The deep learning-based extraction approach takes the original image pixels as input and iterative uncovers hierarchical features. In this way, the decision errors on recognition are minimized. The need for voids is explicitly extracting some image processing-based features that might discard relevant information about image classification. Therefore, the deep learning-based approaches are capable of extracting more complete vein features for recognition, compared to handcrafted approaches. Currently, deep learning-based methods, such as deep neural networks, show high recognition performance because they harness rich prior knowledge acquired by training them on a huge training dataset. However, in finger-vein recognition, it is impossible to capture a lot of vein samples from the same finger, so the training sample of each class is generally limited. For example, the samples from each finger are < 12 in existing databases (Miura et al., 2004, 2007; Das et al., 2019). Especially, there is only one single sample per finger for single sample per person (SSPP) problem when considering their limited storage and privacy policy. Therefore, it becomes particularly intractable for such an identification system with SSPP when within-class information is not available to predict the unknown variations in query samples. Currently, various approaches have been proposed for biometrics identification with SSPP such as face (Wu and Deng, 2016; Wang et al., 2018), palmprint (Shao and Zhong, 2021), and fingerprint (Chatterjee et al., 2017). Similarly, the corresponding finger-vein recognition systems still suffer from the SSPP problem due to the following facts: 1) There is only one enrollment sample in some recognition systems, such as credit card and large-scale recognition, because of the limitation of storage capability. 2) To achieve online identification, the recognition systems usually store one sample per subject to improve processing speed and time. 3) Some users are not willing to cooperatively capture sufficient samples for their personal privacy. Providing one enrollment sample is convenient, simple, and acceptable for users.

As described in previous works, deep learning techniques show a powerful capacity for feature representation, but they generally require sufficient training samples to train a large number of network parameters. Therefore, their learning capacity may have not been well exploited for finger-vein SSPP due to the limited training data of each class (a person or subject) for SSPP. Besides, the deep learning model is easy to suffer from over-fitting on a small amount of dataset. As a result, deep learning-based vein identification approaches may not achieve high performance for finger-vein SSPP.

Ensemble learning aims at combining multiple learners to obtain a more robust representation of the object and is successfully applied for vision tasks such as SAR image category (Zhao et al., 2016), fault diagnosis (Liu et al., 2021), image cluster (Tsai et al., 2014), and human activity recognition (Jethanandani et al., 2020). In addition, some researchers applied it to biometrics, e.g., classification tasks such as fingerprint classification (Zhang et al., 2011b), palm-vein recognition (Joardar et al., 2017), and face recognition (Bhatt et al., 2014; Ding and Tao, 2018). As the features from different learners can achieve a complementary representation for the input image, their combination performs well for identification. To extract enough features from a single finger-vein training sample, in this article, we further research our work on Liu C. et al. (2022) and proposed a deep ensemble learning approach for finger-vein identification.

The rest of the paper is organized as follows. The methodology and significant contributions are listed in Section 2. In Section 3, the proposed method is described in detail. The contract experiment and the results are reported in Section 4. In Section 5, the full research work is concluded for easy reading.

## 2. Proposed method system and our contributions

Motivated by the success of ensemble learning and driven by the SSPP finger-vein recognition, we propose an ensemble deep neural network to learn robust representation from a single sample for SSPP finger-vein recognition. In our work, multiple deep learning classifiers are employed to extract robust features from different feature maps generated from an original input image, and then a robust deep ensemble learning approach is generated by combining all weak classifiers. To further improve the performance of our approach, a shared learning approach is investigated during the training process. Besides, we proposed a learning speed adjustment approach so that all weak classifiers can achieve the best performance at the same time. As each classifier can capture robust features, our ensemble learning approach produces a more complete representation of a finger-vein image for identification. The main contributions of this article are summarized as follows:

(1) This work makes the first attempt at SSPP finger-vein identification. In this work, a deep ensemble learning model is proposed for identification with a single finger-vein training sample. First, we employ three baselines to generate multiple feature maps from an original finger-vein image. With these maps, we train multiple convolutional neural networks (CNNs) in parallel to obtain weak classifiers. Second, all classifiers are combined to obtain an ensemble classifier for vein identification. The experimental results imply that the proposed system achieves state-of-the-art recognition results.

(2) We proposed a shared learning scheme to share representations from multiple feature maps. Generally, the classifier of CNN is easily overfitting for the SSPP problem, so a shared learning scheme is employed to improve their performance. We employ a feature map to train a classifier. In this way, multiple classifiers are obtained to extract features. To improve the performance of classifiers, the knowledge is transferred among classifiers if their input maps are similar. Specifically, for a given classifier, we stop to train it at a fixed number of iterative steps and fine-tune it by relative feature maps. As the knowledge of input maps is exploited by multiple classifiers, combining them can achieve a robust representation of a finger-vein image. The experimental results show that the shared learning scheme can improve the performance of the deep ensemble learning model.

(3) We develop a dynamic speed adjustment scheme to automatically control the learning speed of each classifier. The learning speeds of all classifiers are generally different, so it is difficult for them to achieve the best performance at the same time, which results in poor performance for ensemble classifiers. To solve this problem, a learning speed adjustment approach is proposed to improve the performance of our deep assemble learning approach. For example, we divide the whole training process into several phases. After training a classifier in the current phase, the number of training steps in the next phase is computed by the proposed speed adjustment scheme. In this way, the learning speed of each classifier is adjusted dynamically so that they achieve optimal results at the same time, which enables the ensemble classifier to achieve the best performance for vein identification.

**Figure 1:**
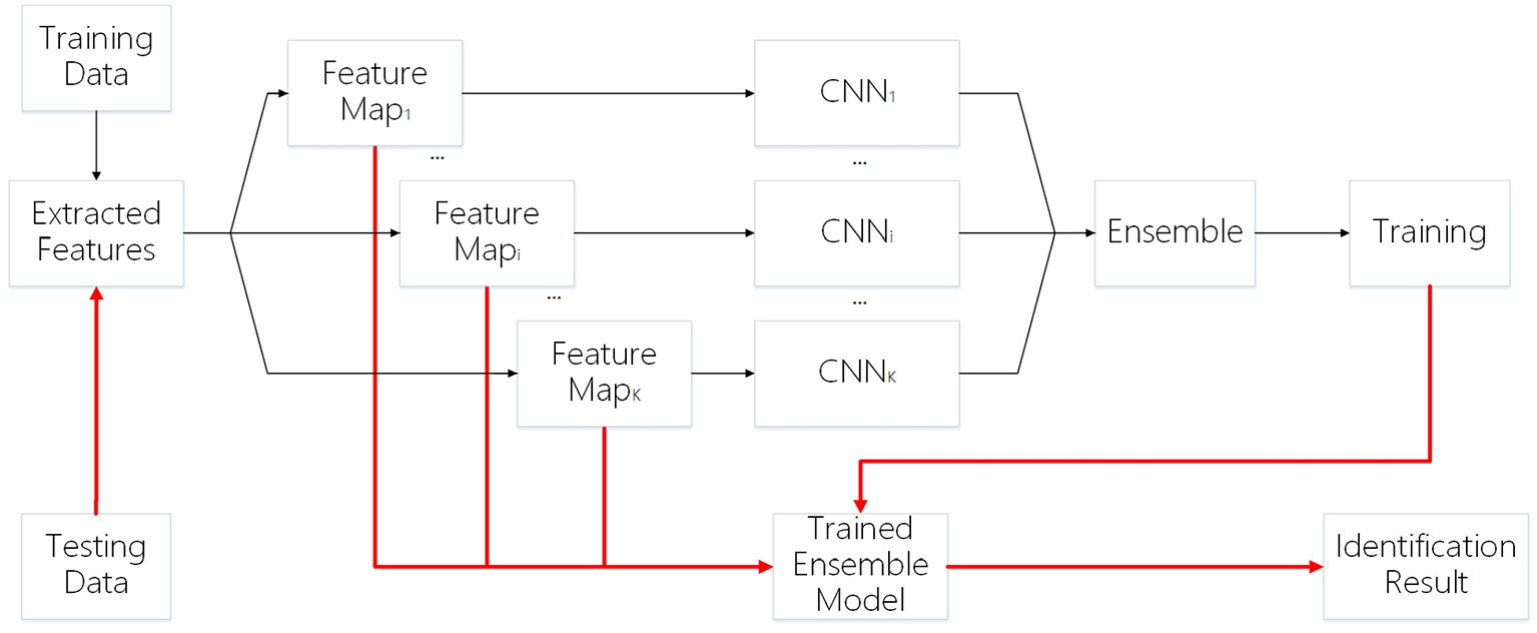
Framework.

The framework of our work is shown in Figure 1. First, we generate feature maps from an input image based on several baselines. With the resulting map and input image, we train several weak classifiers separately. We use CNN models as weak classifiers. Second, we ensemble all classifiers to obtain one ensemble classifier for identification. After training, an input image is subject to prepossessing base on baselines and the resulting maps are taken as the input of our deep learning ensemble mode to compute its probability of being to a class. The testing process is shown in the red line in Figure 1.

## 3. Ensemble learning for SSPP finger-vein recognition

### 3.1. Feature extraction

To achieve robust performance in the SSPP problem, it is necessary to generate multiple feature maps from the single training sample of each class. The feature maps represent different aspects of the object (Felzenszwalb et al., 2010), so their combination can achieve better performance (Li et al., 2018). Here, three baselines (e.g., CNN Das et al., 2019, Gabor filters Zhang et al., 2019a, and LBP Kang et al., 2015) have achieved promising performance for finger-vein recognition, so we employ them to produce feature maps, which are taken as the input of ensemble model for classification.

#### 3.1.1. Finger-vein images

**Figure 2:**
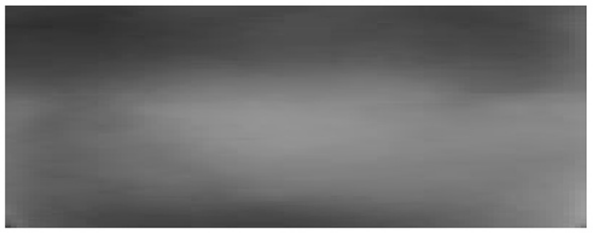
Finger-vein image.

Generally, the vein vessel is difficult to be observed in visible light, but it is captured by near-infrared light with 760 nm wavelength. The vein pattern appears darker than the other regions of the finger because only the blood vessels absorb the infrared rays. Some studies (Kumar and Zhou, 2012) have shown that using extracting vein patterns from vein images achieves promising performance for identification. The finger-vein image sample is shown in Figure 2.

#### 3.1.2. Segmentation

**Figure 3:**
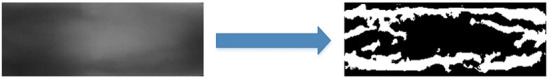
Segmentation of finger-vein image.

As shown in Figure 2, the contrast of the vein pattern in the original finger-vein image is poor, which results in low recognition accuracy. Previous studies showed that image segmentation as a solution has been employed for feature extraction and shows good performance (Miura et al., 2007; Song et al., 2011; Qin and El-Yacoubi, 2017). In recent years, deep learning-based methods have shown a more robust capacity for feature representation compared to handcrafted approaches. So, some researchers bring deep learning-based methods to vein segmentation (Liskowski and Krawiec, 2016; Qin and El-Yacoubi, 2017; Qin et al., 2019; Yang et al., 2019). To train a good weak classifier, the CNN-based model is used to extract robust vein texture patterns, which are input into the weak classifier. First, a CNN-based approach is developed to predict the probability of pixels belonging to veins or backgrounds by learning a deep feature representation. As the finger-vein consists of clear regions and ambiguous regions, several baselines are employed to automatically label pixels as veins or backgrounds in the image's clear regions, thus avoiding the tedious and prone-to-error manual labeling. Then, a CNN is trained to extract the vein patterns from any image region. Second, to improve the performance, an original method based on an FCN was to recover missing finger-vein patterns in the binary image. Figure 3 illustrates the segmentation image from a gray-scale image.

#### 3.1.3. Local binary pattern

The Local binary pattern describes the relationship between the neighborhood points and the corresponding center point, with the features of constant rotation and grayscale. It is widely used to extract finger vein features and shows good performance (Lee et al., 2010, 2011; Rosdi et al., 2011; Yang et al., 2013, 2014; Kang et al., 2015). Therefore, we employ LBP to local information of finger-vein images. The LBP is computed by


(1) $$\begin{array}{l} \begin{array}{c} LBP_{P,R}=\overset{P-1}{\underset{P=0}{\sum }}s\left(g_{p}-g_{c}\right)2^{p}\\ s\left(x\right)=\{\begin{array}{cc} 1, & x>=0\\ 0, & x<0. \end{array} \end{array} \end{array}$$

Where *g*_*c*_ is the gray value of the central pixel, *g*_*p*_ is the value of its neighbors, *P* is the number of neighbors, and *R* is the radius of the neighborhood.

**Figure 4:**
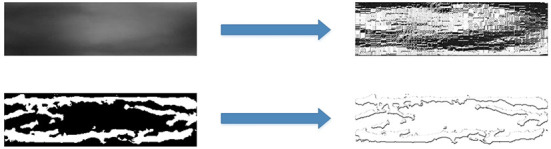
Both original image and segmented image transform by LBP.

In Kocher et al. (2016), the author empirically evaluates different features obtained by using these more recent LBP-related feature extraction techniques for finger-vein spoofing detection. The LBP feature map provides a local representation of a finger-vein image. LBP patterns are extracted from the original image, and segmented image are shown in Figure 4.

#### 3.1.4. Gabor

The Gabor filter is a type of wavelet, it has good time-domain and frequency-domain transform characteristics. Gabor functions are used to construct filters with different scaling directions caused by different parameters (e.g., spatial position, frequency, phase, and direction). Furthermore, the Gabor filter is widely used to capture texture information, and it adapts to extract features from a finger-vein image. In finger vein recognition, there have been more studies using Gabor as a feature, such as Yang et al. (2009), Cho et al. (2012), and Zhang et al. (2019b). The Gabor filter is defined as follows:


(2) $$\begin{array}{l} G=exp\left(-\frac{x^{2}+\gamma^{2}y^{2}}{2\delta^{2}}\right)exp\left(i\left(2\pi \frac{x^{\prime }}{\lambda }+\psi \right)\right) \end{array}$$

where


(3) $$\begin{array}{l} \begin{array}{l} x^{\prime }=xcos\theta +ysin\theta \\ y^{\prime }=-xsin\theta +ycos\theta \end{array} \end{array}$$

In the Gabor function, λ is the wavelength of the cosine factor; θ is the orientation of the normal to the parallel stripes, ψ is the phase offset of the cosine factor, δ is the standard deviation of the Gaussian envelope, and γ is the spatial aspect ratio.

**Figure 5:**
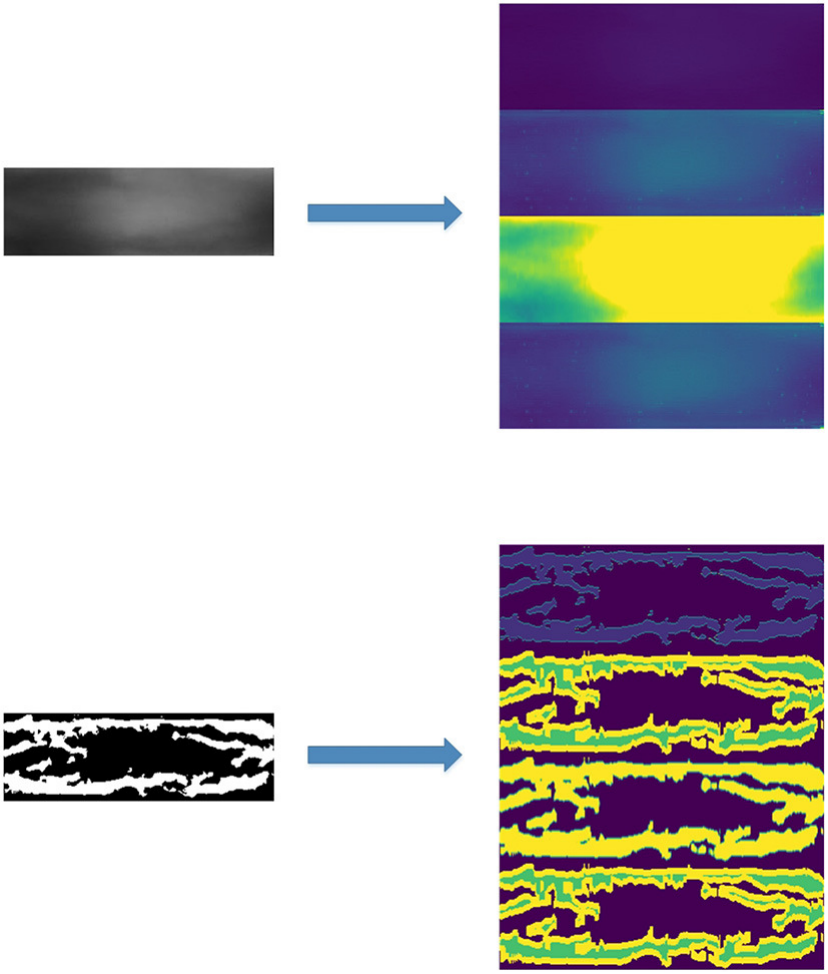
Both the original image and segmented image transform by the Gabor filter.

For the practical application of finger-vein recognition, the real part of the Gabor filter is used. In Zhang et al. (2019a)'s study, the Garbo filter is used to extract texture information of a finger-vein image, and a CNN network is employed to finish the recognition work. The original image and Gabor feature are shown in Figure 5.

**Figure 6:**
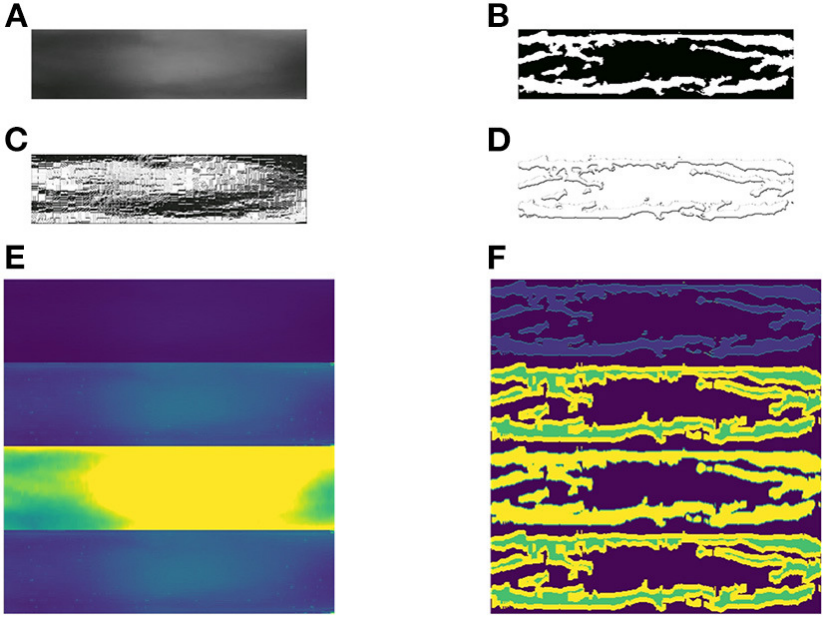
Samples of 6 different feature maps. **(A)** 1st feature map, **(B)** 2nd feature map, **(C)** 3rd feature map, **(D)** 4th feature map, **(E)** 5th feature map, and **(F)** 6th feature map.

We employ the three baselines to extract five different features from the original images. Including the original finger-vein images, there are a total of six feature maps, as shown in Table 1. The six different feature maps are shown in Figure 6.

**Table 1:** Feature maps.

**Input**	**Preprocessing1**	**Preprocessing2**	**Feature map**
Original image	Null	Null	1st feature map
Original image	Segmentation	Null	2nd feature map
Original image	Null	LBP	3rd feature map
Original image	Segmentation	LBP	4th feature map
Original image	Null	Gabor	5th feature map
Original image	Segmentation	Gabor	6th feature map

### 3.2. Convolutional neural networks

The ensemble learning model is constructed from the combination of weak classifiers (Sagi and Rokach, 2018). The choice of a weak classifier affects the performance of the ensemble model. In previous work, CNNs were widely used in finger vein recognition and achieved good recognition performance, such as Wang et al. (2017), Gumusbas et al. (2019), Avci et al. (2019), Gumusbas et al. (2019), and Zhang J. et al. (2019). In this article, we choose the CNN model as the independent weak classifier, and each CNN is trained by one feature map, as shown in Figure 1. Each trained CNN model describes one aspect of the finger vein.

Convolutional neural network is a multi-layer perception network with hidden layers. A traditional recognition model for a classifier can be formulated by minimizing the error function. During training a deep network, the gradient descent method is used to update network parameters. The details of the network structure are shown in Tables 2, 3.

**Table 2:** CNN parameters for the first and the second feature maps.

**Layer type**	**Number of filters**	**Size of Kernel**	**Number of strides**	**Number of padding**	**Dropout**
CL1 (Convolutional layer-1)	64	2 × 2	1 × 1	0 × 0	−
M1 (Max-Pooling Layer-1)	1	2 × 2	2 × 2	0 × 0	−
CL2 (Convolutional layer-2)	64	2 × 2	1 × 1	0 × 0	−
M2 (Max-Pooling Layer-2)	1	2 × 2	2 × 2	0 × 0	−
CL3 (Convolutional layer-3)	64	2 × 2	1 × 1	0 × 0	−
M3 (Max-Pooling Layer-3)	1	2 × 2	2 × 2	0 × 0	−
CL4 (Convolutional layer-4)	128	2 × 2	1 × 1	0 × 0	−
M4 (Max-Pooling Layer-4)	1	2 × 2	2 × 2	0 × 0	−
CL5 (Convolutional layer-5)	256	2 × 2	1 × 1	0 × 0	−
M5 (Max-Pooling Layer-5)	1	2 × 2	2 × 2	0 × 0	−
R1 (ReLu Layer-1)	−	−	−	−	0.5
Softmax layer	−	−	−	−	−

**Table 3:** CNN parameters for the third, the fourth, the fifth, and the sixth feature maps.

**Layer type**	**Number of filters**	**Size of Kernel**	**Number of strides**	**Number of padding**	**Dropout**
CL1 (Convolutional layer-1)	64	2 × 2	1 × 1	0 × 0	−
M1 (Max-Pooling Layer-1)	1	2 × 2	2 × 2	0 × 0	−
CL2 (Convolutional layer-2)	64	2 × 2	1 × 1	0 × 0	−
M2 (Max-Pooling Layer-2)	1	2 × 2	2 × 2	0 × 0	−
CL3 (Convolutional layer-3)	64	2 × 2	1 × 1	0 × 0	−
M3 (Max-Pooling Layer-3)	1	2 × 2	2 × 2	0 × 0	−
CL4 (Convolutional layer-4)	128	2 × 2	1 × 1	0 × 0	−
M4 (Max-Pooling Layer-4)	1	2 × 2	2 × 2	0 × 0	−
CL5 (Convolutional layer-5)	128	2 × 2	1 × 1	0 × 0	−
M5 (Max-Pooling Layer-5)	1	2 × 2	2 × 2	0 × 0	−
CL6 (Convolutional layer-6)	256	2 × 2	1 × 1	0 × 0	−
M6 (Max-Pooling Layer-6)	1	2 × 2	2 × 2	0 × 0	−
R1 (ReLu Layer-1)	−	−	−	−	0.5
Softmax Layer	−	−	−	−	−

In each CNN, the parameters, such as the number of layers and size of the kernel, are different for the different classifiers. In general, the closer relative classifiers have more same parameters. The first feature map and the second feature map are basic features to generate the third feature map, the fourth feature map, the fifth feature map, and the sixth feature map, and a convolutional neural network (CNN) with five layers are employed to extract their feature. As listed in Table 2, the CNN consists of three convolutional layers of 64 kernels with the size of 2 × 2, a convolutional layer of 128 kernels with the size of 2 × 2, and a convolutional layer of 256 kernels with the size of 5 × 5. For the remaining four classifiers, we employ a CNN with six convolutional layers for feature extraction, as shown in Table 3. Specifically, there are 64 kernels with the size of 2 × 2 in the first three convolutional layers and 128 kernels with the size of 2 × 2 in the fourth convolutional layer. The last two convolutional layers include 128 kernels with the size of 5 × 5 and 256 kernels with the size of 5 × 5, respectively. Combing all classifiers, we built an ensemble learning model to identify a subject with a single training sample.

### 3.3. Shared learning

If the feature maps are correlative, we can utilize the knowledge from other weak classifiers to improve the current classifier (Lou et al., 2017). In general, features with higher correlation provides more positive knowledge, which results in the improvement of the weak classifier. So, to achieve the best-shared representations, we compute the similarity among feature maps, which determines the result of shared learning.

#### 3.3.1. Similarity of feature maps

In Zhang et al. (2011a)'s study, a feature similarity index (FSIM) is proposed for the similarity of feature maps.

We compute the FSIM of *feature*_1_(*image*) and *feature*_2_(*image*) to express the correlation of feature 1 and feature 2 of the current image. The FSIM measurement between *feature*_1_(*image*) and *feature*_2_(*image*) are separated into two components, each for PC or GM. The PC value of images can be considered as a dimensionless measure for the significance of a local structure, which is defined in Morrone et al. (1986) and Kovesi (1999). We define 2 features PC and GM to compute the similarity. The PC features *PC*(*image*) is computed as follows:


(4) $$\begin{array}{l} PC\left(image\right)=\frac{E\left(image\right)}{\epsilon +\sum_{n}A_{n}\left(image\right)} \end{array}$$

Where $E\left(image\right)=\sqrt{F^{2}\left(image\right)+H^{2}\left(image\right)}$ and ϵ is a small positive constant, $A_{n}\left(image\right)=\sqrt{e_{n}^{2}\left(image\right)+o_{n}^{2}\left(image\right)}$, $F\left(image\right)=\underset{n}{\sum }e_{n}\left(image\right)$, and $H\left(image\right)=\underset{n}{\sum }o_{n}\left(image\right)$, where *e*_*n*_(*image*) and *o*_*n*_(*image*) the even- and odd-symmetric filters on scale *n* of the image.

The GM feature *G*(*image*) is compute as follows:


(5) $$\begin{array}{l} G\left(image\right)=\sqrt{G_{h}^{2}\left(image\right)+G_{v}^{2}\left(image\right)} \end{array}$$

Where *G*_*h*_(*image*) and *G*_*v*_(*image*) describe the horizontal and vertical directions gradient operators on the image, respectively.

We use *I*_1_ and *I*_2_ to express *image*_1_ and *image*_2_, the FSIM is computed as follows:


(6) $$\begin{array}{l} FSIM\left(I_{1},I_{2}\right)=\frac{\sum_{x\in \Omega }S_{L}\left(I_{1},I_{2}\right)\cdot PC_{m}\left(I_{1},I_{2}\right)}{\sum_{x\in \Omega }PC_{m}\left(I_{1},I_{2}\right)} \end{array}$$

Where Ω means the whole image spatial domain.

The *S*_*L*_ similarity combines the similarity *S*_*PC*_ of PC and the similarity *S*_*G*_ of G:


(7) $$\begin{array}{l} S_{L}\left(I_{1},I_{2}\right)={\left[S_{PC}\left(I_{1},I_{2}\right)\right]}^{\alpha }\cdot {\left[S_{G}\left(I_{1},I_{2}\right)\right]}^{\beta } \end{array}$$

where α and β are parameters used to adjust the relative importance of PC and G features, which is computed as follows:


(8) $$\begin{array}{l} \begin{array}{c} S_{PC}\left(I_{1},I_{2}\right)=\frac{2PC\left(I_{1}\right)\cdot PC\left(I_{2}\right)+T_{1}}{PC^{2}\left(I_{1}\right)+PC^{2}\left(I_{2}\right)+T_{1}}\\ S_{G}\left(I_{1},I_{2}\right)=\frac{2G\left(I_{1}\right)\cdot G\left(I_{2}\right)+T_{2}}{G^{2}\left(I_{1}\right)+G^{2}\left(I_{2}\right)+T_{2}} \end{array} \end{array}$$

where *T*_1_ is a positive constant to increase the stability of *S*_*PC*_, *T*_2_ is a positive constant depending on the dynamic range of GM values.

The *PC*_*m*_ means the maximum value of *PC*(*I*_1_) and *PC*(*I*_2_), which is *PC*_*m*_(*I*_1_, *I*_2_) = *max*(*PC*(*I*_1_), *PC*(*I*_2_)).

After the similarity of different features of a single image is computed, we summarize the similarity of feature maps by the average similarity of all images. We use *F*_*j*_ *S*_*t*_(*F*_*i*_, *F*_*j*_) to express the feature map similarity of feature map i(*F*_*i*_) and feature map j(*F*_*j*_), which is computed as follows:


(9) $$\begin{array}{l} S_{t}\left(F_{i},F_{j}\right)=\frac{\sum FSIM\left(I_{i},I_{j}\right)}{n} \end{array}$$

where *n* is the number of training samples, and *I*_*i*_ and *I*_*j*_ are feature maps in *F*_*i*_ and *F*_*j*_, respectively, but generate from the same finger-vein image.

#### 3.3.2. Shared learning scheme

We divide the training process of the weak classifier into *K* steps, and each step contains *P* epochs. After training the current classifier in each step, the training samples from other feature maps are used to adjust the parameter by shared learning. In this work, we use the feature map similarity *S*_*t*_(*F*_*i*_, *F*_*j*_) to compute the number of epochs used in shared learning *Ec*_*F*_*i*_, *F*_*j*__. The *Ec*_*F*_*i*_, *F*_*j*__ is set as follows:


(10) $$\begin{array}{l} Ec_{F_{i},F_{j}}=2^{\left\lfloor \frac{P}{4}\times S_{t}\left(F_{i},F_{j}\right)\right\rfloor -1} \end{array}$$

where *F*_*i*_ is the feature maps used to train the current classifier and *F*_*j*_ expresses other feature maps.

**Figure 7:**
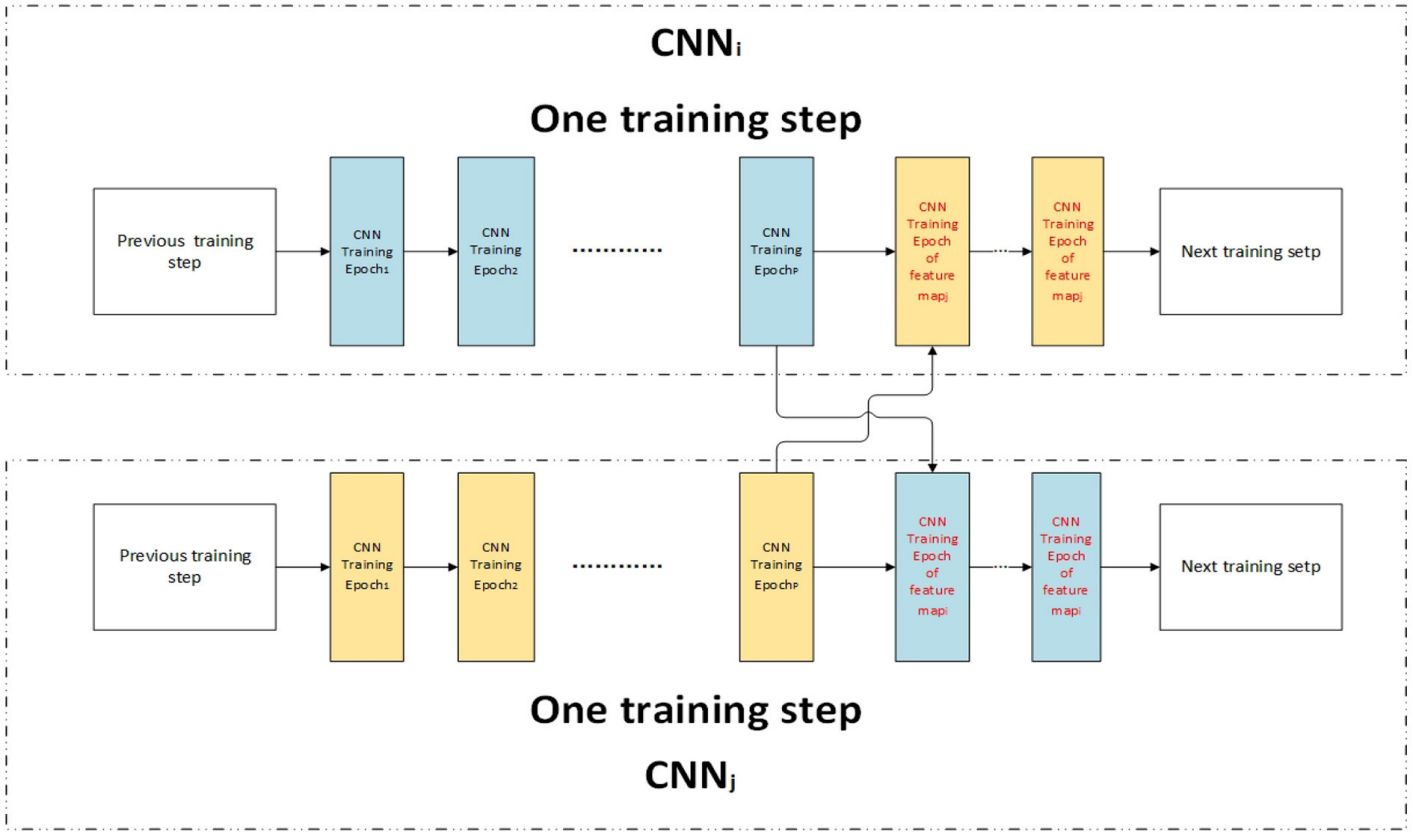
Shared learning by high correlative feature maps.

The performance can be improved by sharing knowledge when high feature correlation (Misra et al., 2016). In the training process, train step *u* is divided into two parts: training the current classifier and shared learning of other classifiers. The shared learning process is followed by the train current classifier process. The network structure is shown in Figure 7. In one train step of *CNN*_*i*_, the data of *feature*_*i*_ are used to train *CNN*_*i*_ by *P* epochs, and then the data of *feature*_*j*_ are used to train *CNN*_*i*_ by *Ec*_*F*_*i*_, *F*_*j*__ epochs. After the data of all high correlative features are used to train *CNN*_*i*_ by shared learning, *CNN*_*i*_ enters the next training step.

### 3.4. Learning speed adjustment

During training weak classifiers, the learning speeds of classifiers are different, because the input data are different. When all weak classifiers achieve their best performance, the ensemble model is best. We propose a learning speed adjustment method to control the learning speeds of weak classifiers. The parameter α is used to adjust the learning speed of each classifier. The whole training process is divided into *K* steps and *P* epochs in each step. The total number of epochs *R* is as follows:


(11) $$\begin{array}{l} R=K*P \end{array}$$

The parameter α_*i, u*_ is used to adjust the number of epochs dynamically for *classifier*_*i*_ of train step *u*. When the classifier had achieved well performance, the number of epochs will decrease in the next step. The α_*i, u*_ is computed as follows:


(12) $$\begin{array}{l} \alpha_{i,u}=\frac{1}{1+e^{-L_{i,u-1}}} \end{array}$$

Where the *L*_*i, u*−1_ is the loss function value after train step *u*−1. To ensure the program runs smoothly, a round down is used to ensure the epoch time is an integer and more than 0. So, the number of epochs *p*_*i, u*_ for train step *u* of *classifier*_*i*_ is as follows:


(13) $$\begin{array}{l} p_{i,u}=\{\begin{array}{ll} 1, & \left\lfloor \alpha_{i,u}\cdot P\right\rfloor <1\\ \left\lfloor \alpha_{i,u}\cdot P\right\rfloor , & other. \end{array} \end{array}$$

### 3.5. Ensemble classifier

There are two problems during ensemble weak classifiers: (1) How to enhance good weak classifiers while weakening poor weak classifiers; (2) How to make all weak classifiers perform best at the same time. The second problem has been discussed in the previous subsection, in this subsection, we discuss the first problem. To tackle the first problem, an access weight can be set to increase the weight of a good classifier and decrease the weight of the pool classifier. The *E*^+^ is computed as


(14) $$\begin{array}{l} E_{i,u}^{+}=\frac{\frac{1}{1+exp\left(-Score_{i,u}\right)}}{\overset{K}{\underset{i=1}{\sum }}\frac{1}{1+exp\left(-Score_{i,u}\right)}} \end{array}$$

where *Score*_*i, u*_ is the close test accuracy of each *classifier*_*i*_ after train step *u*. The *E*^−^ is computed as


(15) $$\begin{array}{l} E_{i,u}^{-}=\frac{\frac{1}{1+exp\left(-L_{i,u}\right)}}{\overset{K}{\underset{i=1}{\sum }}\frac{1}{1+exp\left(-L_{i,u}\right)}} \end{array}$$

where *L*_*i, u*_ is the loss function value after train step *u*.The ensemble weight *W* after train step *u* is updated as follows:


(16) $$\begin{array}{l} W_{i,u}^{*}=\{\begin{array}{ll} \frac{\left|E_{i,u}^{+}-E_{i,u}^{-}\right|}{\overset{K}{\underset{i=1}{\sum }}|E_{i,u}^{+}-E_{i,u}^{-}|}, & E_{i,u}^{+}\geq 0.5\\ 0, & E_{i,u}^{+}<0.5 \end{array} \end{array}$$

where *K* is the number of classifiers. The ensemble weight *W* is updated after each training step. Considering the efficiency of the ensemble model, if the classifier plays poor performance, this classifier could not join the ensemble step. In our model, we use the close test of classifiers to measure their efficiency; if the close test scores more than 0.5, this classifier will join the ensemble step and vice versa.

### 3.6. Structure of training process

Three different approaches are proposed in this article.

#### 3.6.1. Basic approach

**Figure 8:**
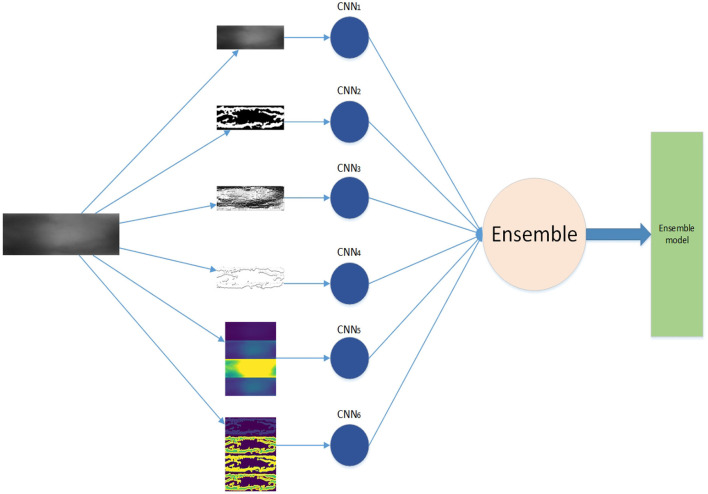
Training structure of basic approach.

The training process of the basic approach is divided into three stages, which are shown as the training part in Figure 1. We extract feature maps as described in Section 3.1, then we train a weak classifier (CNN) as described in Section 3.2, finally, we ensemble all classifiers by vote as described in Section 3.5. Although this approach simply trains the classifier separately, when the ensemble of all classifiers by vote weight during training, both positive contribution and negative effect are considered by employing Equations (14)–(16). To avoid the poor weak classifier play gadfly in the ensemble model, we set a threshold to accept the classifiers and the vote weight by employing Equation (16) in the ensemble model. This structure is shown in Figure 8. The weak classifiers can be trained in parallel.

#### 3.6.2. The shared learning method

**Figure 9:**
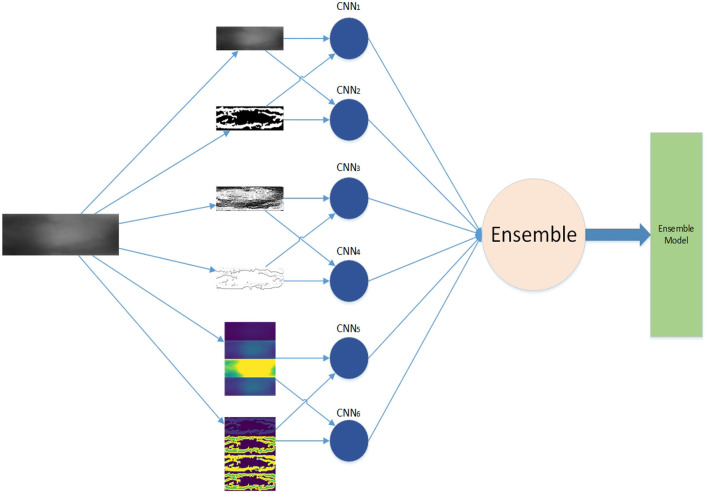
Training structure of the shared learning method.

The shared learning method inherits the basic approach. During training classifiers, other feature maps are used to train classifiers in different ‘levels' in each training step. These ‘levels' correlate by employing Equation (9). The train network structure of shared learning is shown in Figure 9.

#### 3.6.3. Shared learning with adjust learning speed method

**Figure 10:**
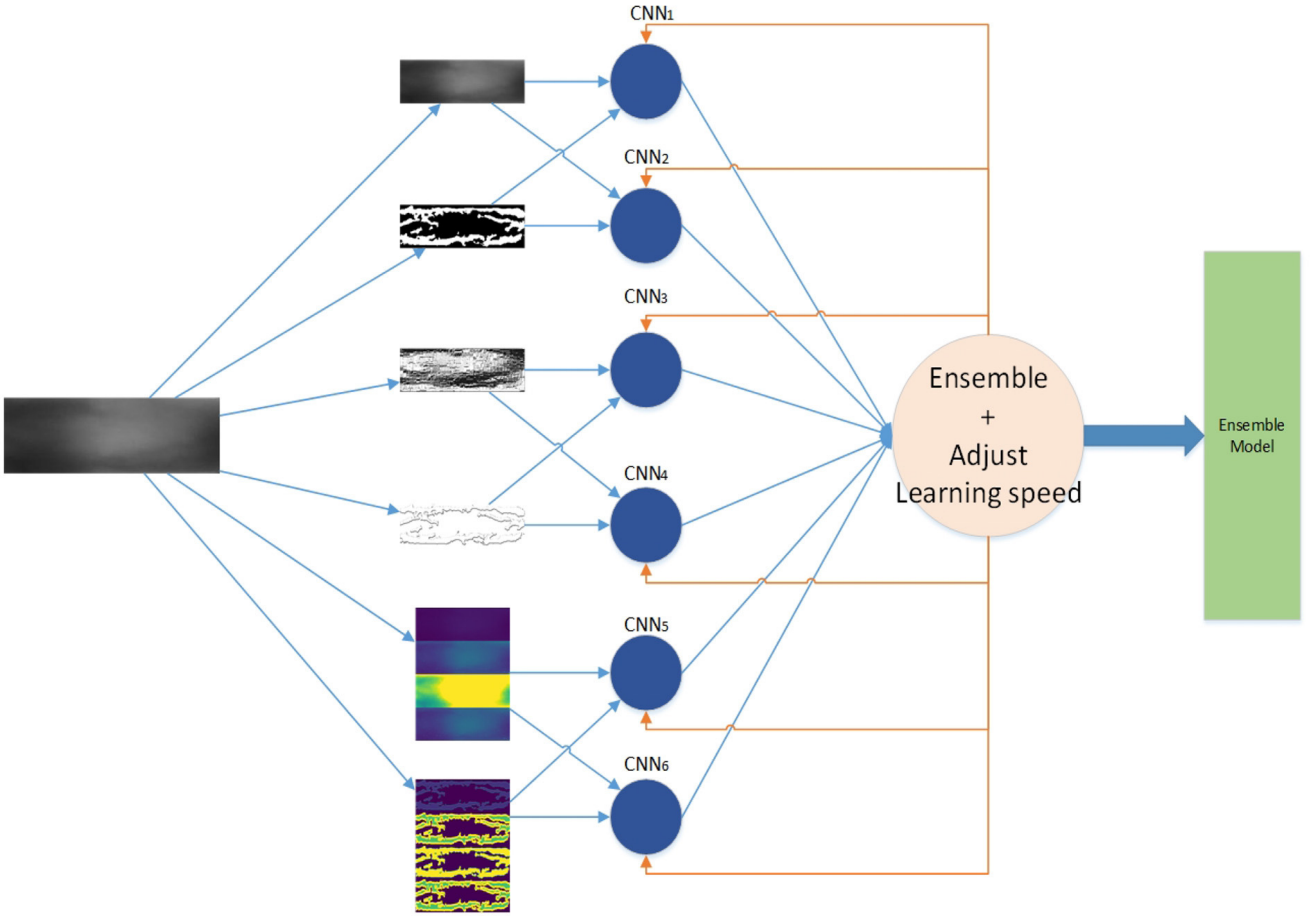
Training structure of the shared learning with adjust learning speed method.

The third method inherits the second one which is shared learning high relative feature maps, in addition, we add the parameter of adjusting learning speed. In this method, the performance of each classifier maintains well after 35 train steps, so the ensemble classifier plays better performance than the other 2 methods proposed before. The parameter of learning speed adjustment by employing Equation (12) to determine the number of epochs of the next train step by employing Equation (13). This parameter is updated at the end of each training step and feedback to train classifiers before the next training step. The train network structure of the shared learning and adjusted learning speed method is shown in Figure 10.

## 4. Experiments

To estimate the performance of our approach, we carry out experiments on two public finger-vein databases, namely the Hong Kong Polytechnic University (HKPU) (Kumar and Zhou, 2012) and the University Sains Malaysia (FV-USM) (Asaari et al., 2014). In experiments, we show the experimental results of each classifier and objective task, respectively. Also, some existing approaches, such as Das et al. (2019), have been employed for finger-vein identification in comparable experiments. All the experiments have been performed in Python 3.7, with a system configuration of 128Gb RAM, Tesla P40 graphics card, and two processors both with Intel(R) Xeon(R) Gold 5118 @ 2.30GHz and Linux version 5.3.0 operating system.

### 4.1. Finger-vein database

In this article, we conduct experiments on the HKPU database and FV-USM database.

**HKPU database**: The HKPU finger-vein image database (Kumar and Zhou, 2012) consists of images from 156 male and female volunteers. It has been acquired between April 2009 and March 2010 using a contactless imaging device at the HKPU campus. It is composed of 3,132 images from 156 subjects, all of them in a BMP format with a resolution of 513 × 256 pixels. In this dataset, about 93% of the subjects are younger than 30 years, and finger-vein images from 105 subjects have been acquired in two separate sessions with a minimum interval of 1 month and a maximum of over 6 months, with an average of 66.8 days. In each session, every subject has provided 6 image samples from the index and middle finger of the left hand. Other 51 subjects have one single session of acquired data. In the experiment, 2,520 images (105 subjects × 2 fingers × 2 sessions × 6 samples) from two separate sessions are employed to test our approach.

**FV-USM database**: The FV-USM database (Asaari et al., 2014) is from University Sains Malaysia. It consists of left and right-hand index and middle fingers' vein images from 123 subjects. Among them, 83 are male and 40 are female, with an age range of 20 − 52 years. All images have been acquired in two different sessions with six images per finger in every session. There are 2,952 images (123 subjects × 2 fingers × 2 sessions × 6 samples) All images are in gray level BMP format with a resolution of 640 × 480 pixels.

The details of both datasets are described in Table 4.

**Table 4:** Finger-vein databases.

**Database**	**Subject**	**Number of fingers**	**Details of fingers**	**Images per finger**	**Sessions**	**Image size**	**Total images**
HKPU	156	2	Left hand index middle finger	12	2	513*256	3,132
FV-USM	123	4	Left and right hand index middle finger	12	2	640*480	5,904

### 4.2. Experiment setup

We aim at solving the finger-vein SSPP problem, so the first image from the first session is selected for training and the three images from the second session are employed for testing. As a result, there are 210 samples in the training set and 630 samples (1,260 fingers × 6 samples) in the test set for the HKPU database. Similarly, there are 246 training samples and 1,476 samples (246 fingers × 6 samples) for the FV-USM database. We train our model on the training set and compute the identification accuracy on the test set to estimate the performance of the proposed method.

In our article, the six feature maps are generated by three baselines (e.g., LBP, Gabor, and CNN), as shown in Table 1, and their parameters are determined based on setting in existing works (Kang et al., 2015; Qin and El-Yacoubi, 2017; Zhang et al., 2019a). For LBP descriptors, the radius is set to 1 which implies that only the one layer around the center pixel and eight pixels around the center pixel are taken into account for the feature's next action. The second baseline e.g., Gabor filter has six scales, namely 7, 9, 11, 13, 15, and 17, the wavelength λ is determined by π/2, and the directions θ are set to 0^*o*^, 45^*o*^, 90^*o*^, and 135^*o*^. For CNN, the network structures and parameters are presented in Tables 2, 3.

### 4.3. Performance impacted by shared learning

In this section, we carry out experiments to verify whether the shared learning scheme improves identification accuracy. As described in Section 3, we proposed a shared learning scheme to learn the knowledge among different feature maps, as shown in Figure 6. Generally, if two feature maps are more relative, the performances of both feature maps are improved by the shared learning scheme. In our article, the similarity/relativity between two feature maps is computed by Equation (9) and further taken as an input of Equation (10) to compute the iteration number of shared learning. As shown in Table 5, the first feature map is highly related to the second feature map, the third feature map, and the fourth feature map on both datasets based on Equation (9). Then, the numbers of interaction steps for there relative shared learning are determined to 4, 1, and 2 by Equation (10), respectively. To achieve shared learning, we employ the second feature map to fine-tune the CNN trained by the first feature map at in four steps, and the resulting CNN model is further trained based on the third feature map, followed by the fourth feature map. In this way, the remaining classifiers are trained for identification. In experiments, we test the six classifiers on both databases mentioned in Section 4.2. For example, there are 210 samples from 210 fingers in the training set and 1,260 samples in the testing set for the HKPU database, and 492 training samples and 2,952 testing samples for the FV-USM database. The identification accuracies of six classifiers with shared learning are listed in Table 6. Also, the performance of each classifier without shared learning is reported in Table 6 for comparison. From the experimental results, we observe that the performance of all classifiers is significantly improved after shared learning. Specifically, the identification accuracy increases by an average of 2% for all weak classifiers, which implies that learning knowledge from other relative feature maps by our shared learning approach can improve the performance of the current classifier. This may be explained by the following facts. Using different features to train weak classifiers can transfer to form a more complementary representation of the original finger-vein image.

**Table 5:** Performance comparisons of shared learning.

**Feature map**	**Shared feature map**	**Feature similarity**	**Epochs**	**Performance**	**Performance without shared learning**	**Database**
1st feature map				67.32%	67.32%	HKPU
1st feature map	2nd feature map	0.64	4	69.91%	67.32%	HKPU
1st feature map	3rd feature map	0.30	4	67.76%	67.32%	HKPU
1st feature map	4th feature map	0.59	4	67.85%	67.32%	HKPU
1st feature map				88.01%	88.01%	FV-USM
1st feature map	2nd feature map	0.68	4	90.06%	88.01%	FV-USM
1st feature map	3rd feature map	0.32	4	87.66%	88.01%	FV-USM
1st feature map	4th feature map	0.55	4	88.28%	88.01%	FV-USM

**Table 6:** Performance comparisons of shared learning in different CNNs.

**Method**	**Database**	

	**HKPU**	**FV-USM**
*CNN*_1_	67.32%	88.01%
*CNN*_1_+ shared learning	70.12%	90.11%
*CNN*_2_	64.68%	87.96%
*CNN*_2_+ shared learning	68.97%	91.25%
*CNN*_3_	54.01%	83.15%
*CNN*_3_+ shared learning	56.36%	85.37%
*CNN*_4_	60.43%	80.02%
*CNN*_4_+ shared learning	62.02%	84.86%
*CNN*_5_	59.31%	85.87%
*CNN*_5_+ shared learning	61.71%	88.07%
*CNN*_6_	76.17%	86.26%
*CNN*_6_+ shared learning	78.42%	89.91%
Basic approach	78.73%	91.03%
Basic approach + shared learning	85.63%	92.31%

The experiment also shows the relation between relativity and performance, as shown in Table 5. The experimental results show that the higher relativity between the two classifiers brings more improvement in identification accuracy. For example, the first feature map achieves the largest improvement in identification performance by transferring the knowledge of the second feature map which has the highest relativity with the first feature map. On the contrary, less improvement is achieved based on the shared learning between the first feature map and the third feature map because the third feature map shows less similarity to the first feature map. The good performance attributes to the fact that the knowledge is easier to be transferred if multiple feature maps have good relativity.

### 4.4. Performance impacted by learning speed

**Figure 11:**
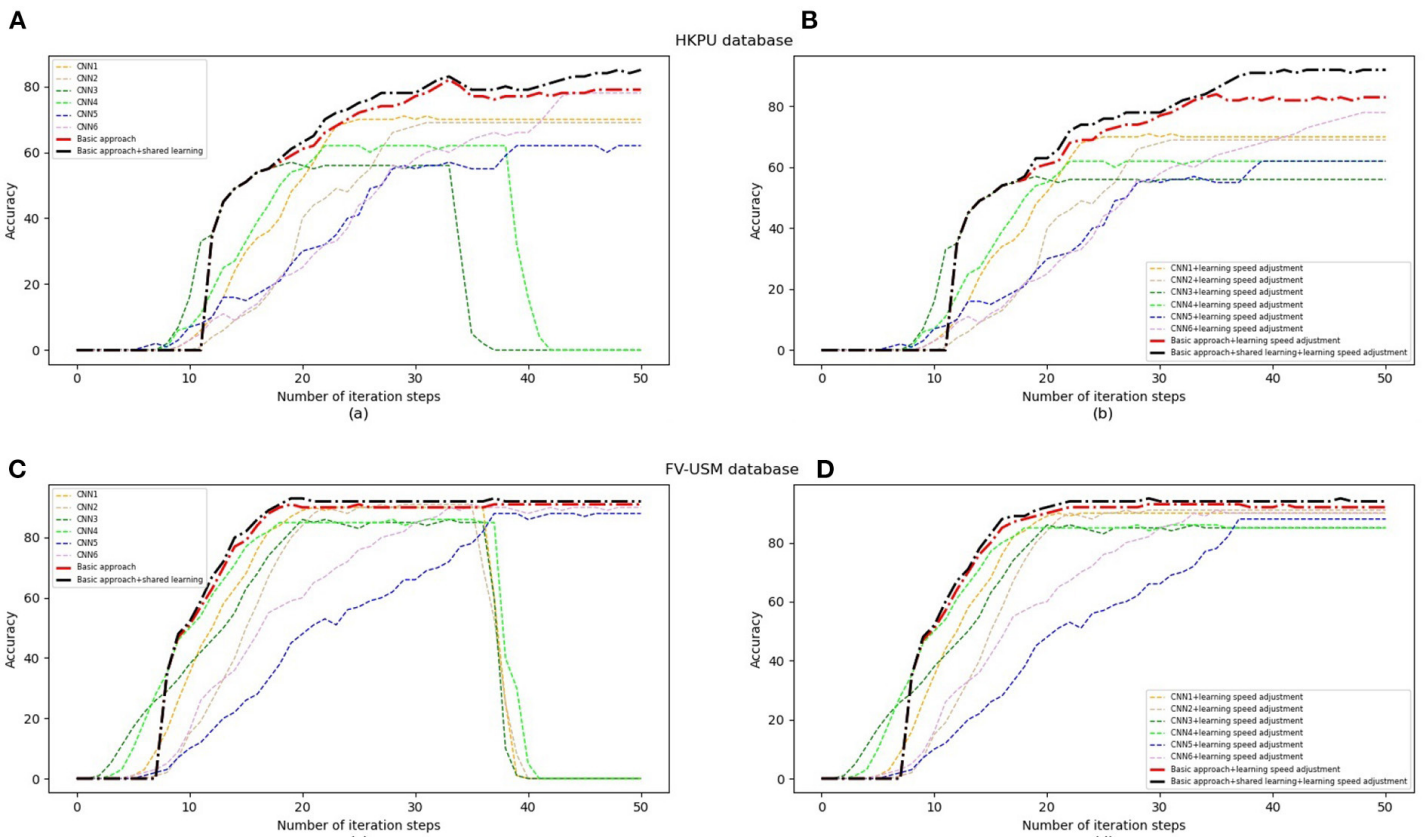
Performance of various classifiers with learning speed adjustment. **(A)** The performance of all our approaches on the HKPU database without adjusting learning speed. **(B)** The performance of all our approaches+adjust learning speed on the HKPU database. **(C)** The performance of all our approaches on the FV-USM database without adjusting learning speed. **(D)** The performance of all our approaches+adjust learning speed on the FV-USM database.

As described in the subsection, our approach is proposed by combining six weak classifiers. In general, the learning speed of each classifier is different. Therefore, the object task can achieve the highest identification accuracy only if each classifier achieves optimal performance at the same time. In this section, the experiments are carried out to evaluate how to impact the performance of object tasks by employing Equation (12) and Equation (13) to adjust the learning speed of each classifier. Figures 11A, B show the identification accuracy of six classifiers and our approach to the HKPU database before and after adjusting the learning speed. In Figure 11A, the performance of all approaches is improved when the number of iteration steps is < 30. However, two classifiers achieve significant degradation in identification accuracy after 35 iterations. The reason is that the two classifiers have higher speed than the remaining classifiers so they cannot achieve optimal performance at the same time. Therefore, it is difficult for object tasks to achieve good performance. In contrast, the learning speed of all weak classifiers is adjusted dynamically, they show the best performance after about 50 iterations (as shown in Figure 11B), which brings the improvement of the objection classifier. Therefore, our approach achieves higher identification accuracy after employing Equation (12) and Equation (13) for learning speed adjustment. The experiments on FV-USM (as shown in Figures 11C, D) show consistent trends that all weak classifiers can achieve optimal performance at the same time (about 40 iterations). In addition, observed training curves (Figures 11A–D), we see that all approaches show good stability after employing the learning speed adjustment scheme.

Table 7 lists the identification accuracy of the basic model, basic approach + shared learning, basic approach + speed adjustment, and basic approach + shared learning + speed adjustment on the HKPU database and FV-USM database. The experimental results have shown that the basic model and basic approach + shared learning achieve a significant improvement in identification accuracy after adjusting learning speed by Equation (13). For example, the basic approach + shared learning and basic approach + shared learning + learning speed adjustment achieve 82.60% and 92.10% identification accuracy on HKPU database and FV-USM database, respectively, and improves identification accuracy by about 7% and 1% compared to basic approach + shared learning. The experimental results in Table 7 imply that our learning speed adjustment scheme is effective to improve the performance of object tasks for an ensemble learning problem.

**Table 7:** Performance comparisons of the adjust learning speed method.

**Method**	**Database**	

	**HKPU**	**FV-USM**
Basic approach	78.73%	91.03%
Basic approach + shared learning	85.63%	92.31%
Basic approach + learning speed adjustment	82.6%	92.1%
Basic approach + shared learning	92.11%	94.17%
+ learning speed adjustment		

### 4.5. Performance comparisons

In this section, we compare our approach with existing approaches to evaluate the performance of our method in terms of improving identification accuracy. In experiments, state-of-art methods, such as Das et al. (2019), Kumar and Zhou (2012), and Song et al. (2011), are employed for finger-vein identification with a single training sample per finger. As described in the section, we select the first image for training and six images collected in the second session for testing. As a result, there are 210 training samples and 1,260 testing samples for the FV-USM database and 496 training samples and 2,952 testing sample for the FV-USM database. The identification accuracies of various approaches have been listed in Table 8 for comparison. From the experimental results, we can observe that the proposed approach (basic approach + shared learning + learning speed adjustment) outperforms the existing approaches and achieves the highest identification accuracy, e.g., 92.11 and 94.17% on the HKPU database and FV-USM database, respectively. Also, we observed that the hand-crafted approaches (Song et al., 2011; Kumar and Zhou, 2012; Qin et al., 2013), considered our work achieve < 91.00% identification accuracy on both databases. Such a poor performance may be attributed following facts: (1) The handcrafted segmentation-based approaches assume that the cross-sectional profile of a vein pattern shows a valley (Miura et al., 2007) or line-like texture (Miura et al., 2004) and proposed various mathematical models to extract vein patterns. However, the vein pixels create more complex distributions instead of the valley or straight lines and the pixels in the non-vein region also show valley or line-like attributes, so their performance is limited. (2) The handcrafted segmentation-based approaches usually match vein networks stored in testing samples and enrolment samples for identification. Therefore, it is difficult for such a matching scheme to achieve good performance when there are larger variations such as rotation, scaling, and translation between two images. Compared to handcrafted segmentation-based approaches, the deep learning-based approach (Qin and El-Yacoubi, 2017) is capable of extracting robust vein networks from a law image because it harnesses rich prior knowledge from huge training samples without any assumption. Therefore, the deep learning-based approach (Qin and El-Yacoubi, 2017) achieves better performance, e.g., 91.75 and 92.29%, identification accuracies on both databases. Similarly, its matching scheme is not robust for image samples with large rotation and translation variations. As a solution, a convolutional neural network (Das et al., 2019) is proposed to extract high-level features instead of a vein network by representation learning that is objectively related to vein identification and achieves promising performance. However, it does not perform well for the SSPP problem. This is explained by the following facts. Deep learning-based approaches generally require a large number of training samples to estimate a usually huge number of deep network parameters. In the SSPP configuration, there is only one training sample for each class, so the learning capacity becomes weak and subject to string overfitting, which leads to low identification performance. Ensemble learning is a solution to the SSPP problem because it can exploit the knowledge from different feature maps to improve identification accuracy. Therefore, our basic approach + shared learning achieves 85.63 and 92.31% accuracies on both datasets, which are further improved to 92.11 and 94.17% by adjusting the learning speed of all weak classifiers. Such a good performance may be attributed to these facts. Each classifier includes different discriminate features. As shown in Table 5, the relative feature maps can make a positive contribution to classification, so the knowledge related to classification is transferred among feature maps by shared learning, which effectively improves the feature representation capacity of object tasks. Meanwhile, the learning speed adjustment ensures each classifier achieves the best performance at the same time so that the object task performs the best representation for vein identification.

**Table 8:** Performance comparisons with state-of-art methods.

**Method**	**Database**	

	**HKPU**	**FV-USM**
Rig (Das et al., 2019)	82.19%	91.75%
MC (Miura et al., 2007)		90.34%
RLT (Miura et al., 2004)		78.28%
Qin (Qin and El-Yacoubi, 2017)	91.75%	92.29%
Gabor filters (Kumar and Zhou, 2012)	77.78%	86.96%
Difference-curvature (Qin et al., 2013)	73.97%	83.91%
Mean-curvature (Song et al., 2011)	88.89%	87.01%
Region-growth (Qin et al., 2011)	82.29%	86.62%
Basic approach + shared learning	85.63%	92.31%
Basic approach + shared learning	92.11%	94.17%
+ learning speed adjustment		

## 5. Conclusion

In this article, the authors proposed a new deep ensemble learning approach for SSPP finger-vein recognition. Multiple improvements have been made in this work. A schema of generating multiple feature maps from a single training image is proposed to enhance the performance of the training section. A shared learning schema is applied to the classifier training section. A new learning speed adjustment approach is proposed to improve the performance of the weak classifiers. With a solid comparative experiment, it is very convincing that the proposed method is an outstanding solution for the SSPP finger-vein recognition problem. First, we generate feature maps from training images. Second, we propose a shared learning scheme during the training of weak classifiers. Third, an approach is proposed to adjust the learning speed of weak classifiers. In the experimental part, we compare the performance of our method with the state-of-art method. The final result of the whole model can achieve state-of-the-art recognition results.

## Data availability statement

Publicly available datasets were analyzed in this study. This data can be found at: The Hong Kong Polytechnic University Finger Image Database, Version 1.0: http://www4.comp.polyu.edu.hk/~csajaykr/fvdatabase.htm Vein USM (FV-USM) Database: drfendi.com/fv_usm_database/.

## Author contributions

CL contributed to the conception of the study, performed the experiment, and wrote the manuscript. HQ performed the data analyses and wrote the manuscript. QS contributed significantly to analysis and manuscript preparation. HY performed the experiment. FL helped significantly to check manuscript preparation. All authors contributed to the article and approved the submitted version.
